# Supplementary material for: The Potential Geographic Distribution of Bactrocera minax and Bactrocera tsuneonis (Diptera: Tephritidae) in China
Source: Insects. 2025 Dec 16;16(12):1277. doi: 10.3390/insects16121277 (PMC12733963; doi:10.3390/insects16121277)
Supplement: Supplementary file 1 [file insects-16-01277-s001.zip › insects-3944867-supplementary.pdf]

Figure legends:

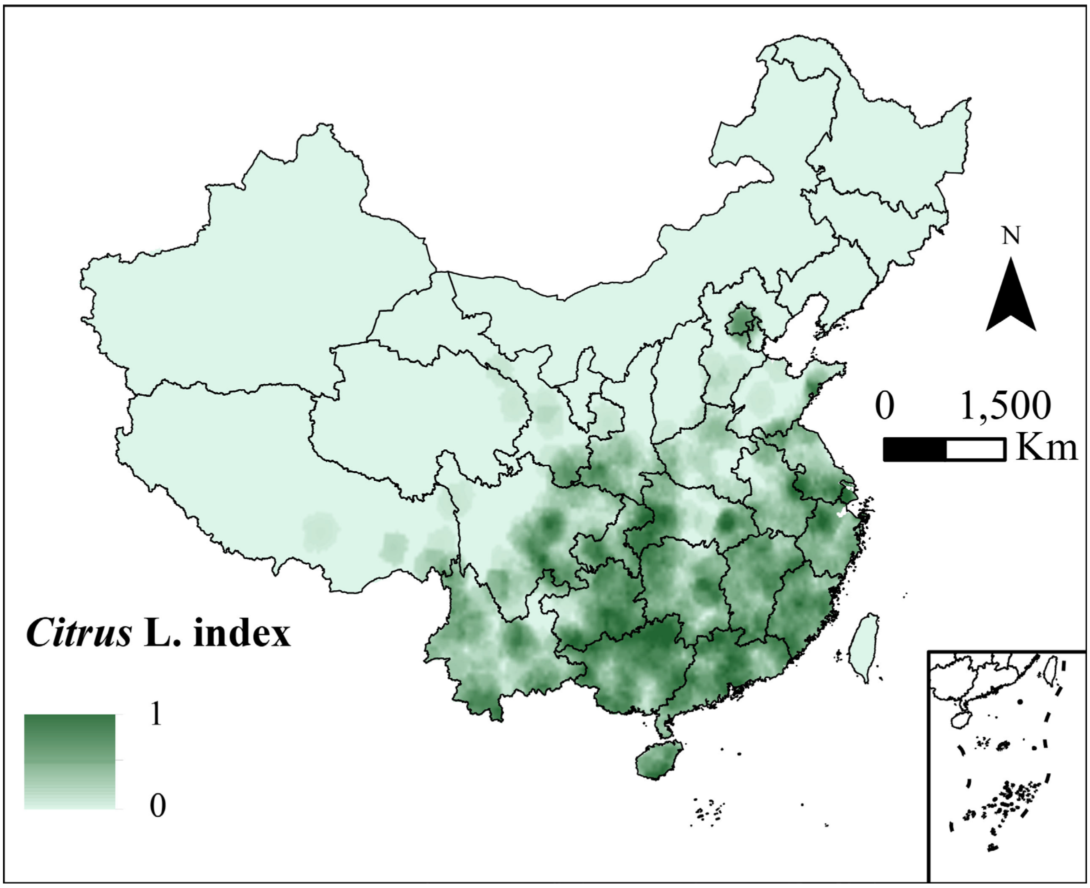

Figure S1. image of *citrus* L. index in China.

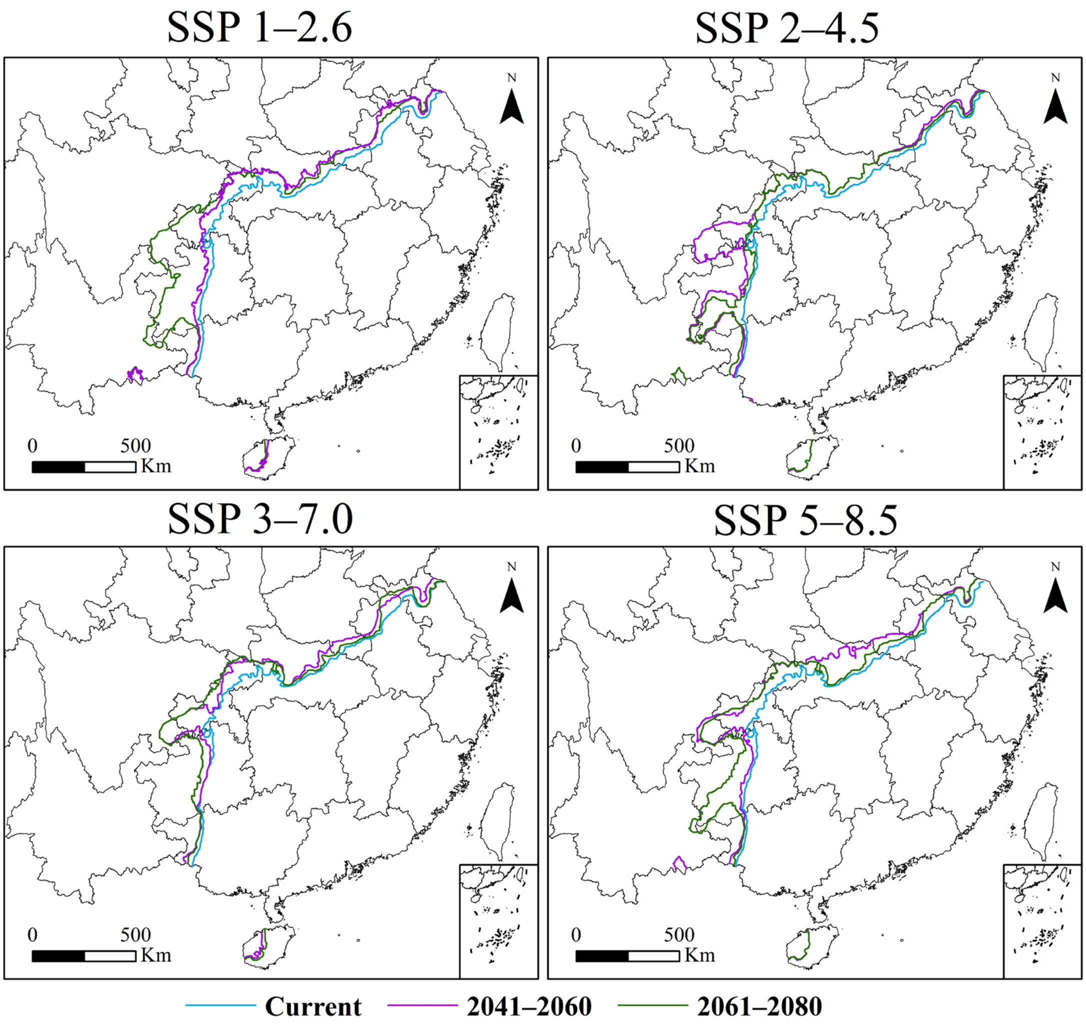

Figure S2. 20 mm contour of BIO14 under four shared socioeconomic pathways (SSP).
